# Viral load suppression among pregnant and breastfeeding women living with HIV in Ghana: a prospective longitudinal study

**DOI:** 10.1186/s12879-026-13282-x

**Published:** 2026-04-14

**Authors:** Joycelyn Assimeng Dame, Kira J. Nightingale, Promise Sefogah, Islynn Aggrey, Derartu Ahmed, Adwoa K. A. Afrane, Elizabeth D. Lowenthal

**Affiliations:** 1 Department of Child Health, University of Ghana Medical School, College of Health Sciences, Accra, Ghana; 2 Department of Biostatistics, Epidemiology and Informatics, University of Pennsylvania Perelman School of Medicine, Philadelphia, PA USA; 3 Department of Obstetrics and Gynaecology, University of Ghana Medical School, College of Health Sciences, Accra, Ghana; 4 Department of Child Health, Korle Bu Teaching Hospital, Accra, Ghana; 5 Global Health Center, Children’s Hospital of Philadelphia, and Department of Pediatrics, University of Pennsylvania Perelman School of Medicine, Philadelphia, USA

**Keywords:** Peripartum, HIV, Vertical transmission, Dolutegravir, Viral suppression, 12 months post-delivery

## Abstract

**Background:**

The post-delivery period is a critical window for sustaining maternal viral suppression (VS) to prevent vertical transmission (VT) of HIV during breastfeeding. Despite Ghana’s national scale-up of dolutegravir-based antiretroviral therapy, limited data exist on viral load suppression and infant HIV outcomes in the year post-delivery. We aimed to determine the proportion of women who achieved and maintained a suppressed viral load (VL) (≤ 1000 copies/mL) and an undetectable VL (< 20 copies/mL) at 12 months post-delivery and examine associations between maternal characteristics and VL categories.

**Methods:**

We conducted a prospective longitudinal cohort study among 120 women living with HIV receiving ART across two tertiary hospitals in Ghana. Women were enrolled during late pregnancy or early post-delivery and followed up at 6 weeks and 3-, 6-, and 12-months post-delivery, during which maternal VL was monitored. Infant HIV status was documented at 6 weeks and 9 months in accordance with national guidelines. Our co-primary outcomes were VL suppression and undetectable VL at 12 months.

**Results:**

The median maternal age was 31.0 years (28.0, 36.3), and nearly half of the participants had attained only primary-level education (58/120; 46.7%). At 12 months post-delivery, 65 participants (54.2%) had documented VS, and out of this, 41 (34.2%) had an undetectable VL. Two cases of VT of HIV were documented: one at 6 weeks (maternal VL 669 copies/mL) and another at 6 months (maternal VL 64 copies/mL), underscoring the transmission risk even with low-level viremia. VL data were missing among 49 participants (40.8%) despite follow-up efforts. Women on the tenofovir/lamivudine/dolutegravir regimen were more likely to achieve suppression than were those on the regimen without an integrase inhibitor, *p* = 0.04.

**Conclusions:**

Just over half of women achieved VS at 12 months post-delivery. VT occurred even at low levels of maternal viremia, underscoring the importance of sustained VL monitoring and optimisation of antiretroviral therapy during the post-delivery period.

**Clinical trial number:**

Not applicable.

**Supplementary Information:**

The online version contains supplementary material available at 10.1186/s12879-026-13282-x.

## Background

In the absence of any intervention, the risk of vertical transmission (VT) of HIV during pregnancy, labour, delivery, or breastfeeding ranges from 15% to 45% [1]. The introduction of lifelong maternal antiretroviral therapy (ART), referred to as Option B+, has made the elimination of HIV a feasible public health goal [2].

Achieving such elimination requires early identification of pregnant women living with HIV (WLHIV), prompt ART initiation, and sustained VS throughout pregnancy and breastfeeding. In 2024, the adult HIV prevalence in Ghana was 1.49%, with 68% of people living with HIV aware of their status; among those diagnosed, 69% were receiving antiretroviral therapy, and 90% of those on treatment were virally suppressed [3]. Despite high prevention of mother-to-child transmission coverage, with 99.3% of pregnant WLHIV receiving ART, these indicators remain below the UNAIDS 95–95–95 targets, and VT continues to occur, highlighting persistent gaps in sustained VS during pregnancy and breastfeeding [3]. In 2024, the reported VT rate at 6 weeks was 2.64%, with a final transmission rate of 9.41%, which includes the breastfeeding period [3].

Breastfeeding is a vital child survival strategy in low-resource settings due to its protective effects against malnutrition and early infant mortality. In Ghana, WLHIV who are on ART are advised to exclusively breastfeed for the first six months, with continued breastfeeding up to one year [4]. Ghana’s policy reflects a cautious balance between maximising the benefits of breastfeeding and minimising the risk of postnatal HIV transmission in settings where maternal ART adherence and routine VL monitoring may not be consistently ensured. Nonetheless, maternal viremia during breastfeeding is strongly associated with post-delivery HIV transmission [5].

The pharmacokinetic properties of dolutegravir—including strong placental and breastmilk penetration—make it particularly well suited for perinatal HIV prevention [6, 7]. However, suppression depends on maternal adherence and access to care. Despite the international scale-up of more potent ART regimens, current data on VS during the post-delivery period among breastfeeding WLHIV are limited.

In Ghana, VL testing is recommended for pregnant women at 34–36 weeks’ gestation, irrespective of when ART was initiated, to identify those at highest risk. Post-delivery VL monitoring follows the same schedule as for the general population, with testing recommended 6 months after ART initiation and annually thereafter if VS is achieved [4]. The guidelines do not specifically address the unique needs of breastfeeding women who are both at higher risk of viral non-suppression and whose viremia poses a risk of transmission to the infant. Many mothers may go unmonitored throughout the breastfeeding period if they have a suppressed VL near delivery. Modelling studies in sub-Saharan Africa suggest that fewer than 30% of women receive VL monitoring during the antenatal or post-delivery period under non-specific guidelines [8].

The post-delivery period also poses unique challenges for retention and adherence [9]. A systematic review and meta-analysis of 51 studies involving over 20,000 pregnant women—mainly from the United States, Kenya, South Africa, and Zambia—estimated that 73.5% (95% CI: 69.3–77.5%) had adequate ART adherence (> 80%). Adherence was significantly greater during the antepartum period (75.7%, 95% CI: 71.5–79.7%) than during the post-delivery period (53.0%, 95% CI: 32.8–72.7%; *P* = 0.005). Barriers to adherence included physical, economic, and emotional stresses, depression (especially post-delivery), alcohol or drug use, and the frequency of ART dosing or pill burden [10].

This study aimed to determine the proportion of women with VL suppression (VL ≤ 1000 copies/mL) or undetectable VL (VL < 20 copies/mL) at 12 months post-delivery and to examine the associations between maternal characteristics and VL suppression in Ghana. Our findings aim to inform improvements in maternal care and guide policies that accelerate progress toward the elimination of HIV in Ghana and similar settings.

## Methods

### Study design and site

We conducted a prospective longitudinal cohort study of WLHIV receiving care at two tertiary hospitals in Ghana between Jul 1 2023 and Dec 31 2024. The study sites were Korle Bu Teaching Hospital (KBTH) and Greater Accra Regional Hospital (GARH).

KBTH is the largest tertiary referral and teaching hospital in Ghana, located in Accra. Its obstetric unit performs over 10,000 deliveries annually and had an antenatal care (ANC) attendance of 20,247 in 2024. The hospital also hosts the Fevers Unit, the largest adult HIV clinic in Accra. GARH is a regional referral hospital with approximately 7,000 annual deliveries and 36,066 ANC attendances in 2024. Both hospitals provide comprehensive obstetric and HIV care services. HIV services are integrated within ANC. After delivery, mothers are followed in postnatal clinics for the first six weeks, then transitioned to HIV clinics for ongoing care, while infants continue follow-up in paediatric HIV clinics. Both hospitals follow national guidelines for ART initiation, including tenofovir, lamivudine and dolutegravir (TLD) for adults and 12 weeks of zidovudine and nevirapine prophylaxis for HIV-exposed infants. While most adults are on TLD, not all are on this regimen. In such cases, the decision to use an alternative regimen is typically clinical, based on factors such as ongoing success with a different regimen. However, all those initiating treatment for the first time are started on TLD. In 2024, HIV prevalence in the Greater Accra Region, where both study sites are located, was 1.80%, exceeding the national average [3]. Additionally, more than 90% of women in the region practice exclusive breastfeeding during the first six months of life.

### Study population

Participants were enrolled from the obstetric units at KBTH and GARH during the third trimester of pregnancy. We also enrolled post-delivery WLHIV and their infants who were attending the paediatric HIV clinics and were not previously captured during pregnancy. We recruited women up to 3 months post-delivery to avoid excluding those who may have missed earlier post-delivery visits and to allow assessment of VL status during the post-delivery period. Mothers and their infants were actively followed at 6 weeks and 3-, 6-, and 12-months post-delivery. Individuals under 18 years of age who were not considered mature minors, those who did not intend to receive follow-up care at our study sites, and individuals who were unable to communicate in English or in one of the three main local languages (Akan, Ga, or Ewe) were excluded. Potential study participants were identified during routine clinic visits and recorded in screening logs in the order in which they were encountered. Eligibility was assessed sequentially according to these logs, and those meeting the inclusion criteria were consecutively enrolled. Written informed consent was obtained prior to participation.

Sample size was determined to estimate the prevalence of VS and undetectable VL with acceptable precision using the standard formula for a confidence interval for a single proportion [11]. A prior study reported that 62% of pregnant and breastfeeding women were virally suppressed, and 53% had an undetectable VL [12]. These estimates were used to inform our sample size calculations. With a sample size of 150 participants, the corresponding 95% confidence interval (CI) for a prevalence of 62% would be 0.54–0.70, and for a prevalence of 53% would be 0.45–0.61. With a minimum sample size of 100 participants, the 95% CIs would widen to approximately 0.53–0.72 for VS and 0.43–0.63 for undetectable VL. We therefore aimed to enroll 100–150 participants, balancing feasibility, anticipated loss to follow-up, and precision of the primary endpoint estimates.

### Data collection and study procedures

Before commencing the main study, the data collection tool was pilot-tested among 10 WLHIV who met the study’s eligibility criteria. The pilot participants were recruited from the same facility as the main study population but were not included in the final sample. The pilot aimed to assess the clarity, relevance, and ease of administration of the data collection tool, and the feedback obtained was used to refine and improve it. At enrollment, contact information, including primary and secondary phone numbers, was collected to facilitate follow-up. Study staff made reminder calls before scheduled visits and contacted participants who missed appointments to encourage continued participation. For individuals who became LTFU, active efforts were made to re-establish contact and re-engage them in the study. Participants were called repeatedly over several weeks, and transportation support was offered to facilitate return visits when needed. These active follow-up measures were implemented to minimise attrition and ensure data completeness, while acknowledging the practical challenges of participant retention in this setting. At the study entry visit, a structured maternal interview was conducted, along with a medical record review. Data were collected using interviewer-administered tools delivered in English or translated into a local language by trained multilingual staff. Enrolment data included sociodemographic characteristics, parity, timing of HIV diagnosis, ART regimen, disclosure status, and self-reported adherence assessed using a validated three-item scale [13]. Depressive symptoms were screened at 6 weeks postpartum using the Edinburgh Postnatal Depression Scale [14]. Follow-up visits reassessed maternal adherence, breastfeeding practices, maternal health, and infant outcomes. Both infants were followed in cases of twin births. Clinical information abstracted from records included current ART regimen, maternal VL results, infant prophylaxis (type and duration), and infant HIV status. VL testing was performed using the Roche Cobas 6800 system (lower limit of detection: 20 copies/mL). This enhanced VL monitoring exceeded routine care and enabled closer assessment of virologic outcomes during the post-delivery period. Infant HIV status was determined using DNA PCR at 6 weeks and 9 months as per national guidelines, with confirmatory testing for positive results. For any breastfeeding infants whose mothers were documented as having VL > 1000 copies/ml, we initiated daily nevirapine prophylaxis, as per local guidelines. Women were withdrawn from the study in cases of stillbirth, early infant death, or confirmed vertical HIV transmission. Excluding these pairs ensured the cohort represented the appropriate risk set for the study question while maintaining ethical and practical considerations.

All laboratory samples from both sites were processed centrally at the KBTH laboratory. The study’s data collection tool is included as supplementary file 1.

VL was classified in two ways: as a binary variable with a VL ≤ 1000 copies/ml defined as suppressed [15] and as a three-level variable with < 20 copies/ml classified as undetectable [15], 20-1000 copies/ml classified as low-level viremia (LLV), and VL > 1000 copies/ml classified as viremia. Lost to follow-up (LTFU) was defined as the absence of a documented maternal VL result at 11–14 months post-delivery for participants who could not be reached by phone and remained untraceable in the national database for more than 3 months after their scheduled appointment. Education level was defined as the highest level of formal education attained - none, primary, secondary, or tertiary.

### Outcomes

The co-primary outcomes were the proportions of women with VS and with an undetectable VL at the 12-month timepoint. Our secondary objectives were to determine the maternal characteristics associated with VL outcomes at 12 months, to describe the VL across all time points and to determine the infant HIV transmission outcome.

### Data management and analysis

The data were entered into REDCap [16] by trained study staff. Medical records were reviewed to validate self-reported information whenever possible. Each blood sample was assigned a unique code to maintain participant confidentiality.

For maternal characteristics, mothers of twins were included only once in the analysis, whereas both twin infants were included in the analysis of infant outcomes. Sociodemographic and clinical characteristics of the cohort were summarised using medians and interquartile ranges (IQR) for continuous variables and frequencies and percentages for categorical variables. Categorical maternal characteristics, including ART regimen, disclosure of HIV status, educational level, and employment status, were compared across VL status groups using the chi-square or Fisher’s exact test, depending on cell sizes. Continuous variables such as maternal age, parity, cost of travel to the hospital, and depression scores were assessed for normality using visual assessment and Shapiro-Wilk tests. All continuous variables were found to be non-normally distributed, and groups were therefore compared using the Kruskal–Wallis test. All analyses were two-sided, and a p-value of ≤ 0.05 was considered statistically significant. A post-hoc time-to-event analysis was run assessing the time to first undetectable VL using a Kaplan-Meier curve. Only participants enrolled during pregnancy were included in this analysis. Given the known association between ART use and viral suppression, Kaplan-Meier curves stratified by timing of initiation of current ART regimen (during pregnancy versus prior to pregnancy) was performed. Differences between the two groups were assessed using a Cox proportional hazards model, after the assumption of proportional hazards was confirmed to be met. Data analysis was performed using R version 4.4.2 [17].

## Results

**Fig. 1:**
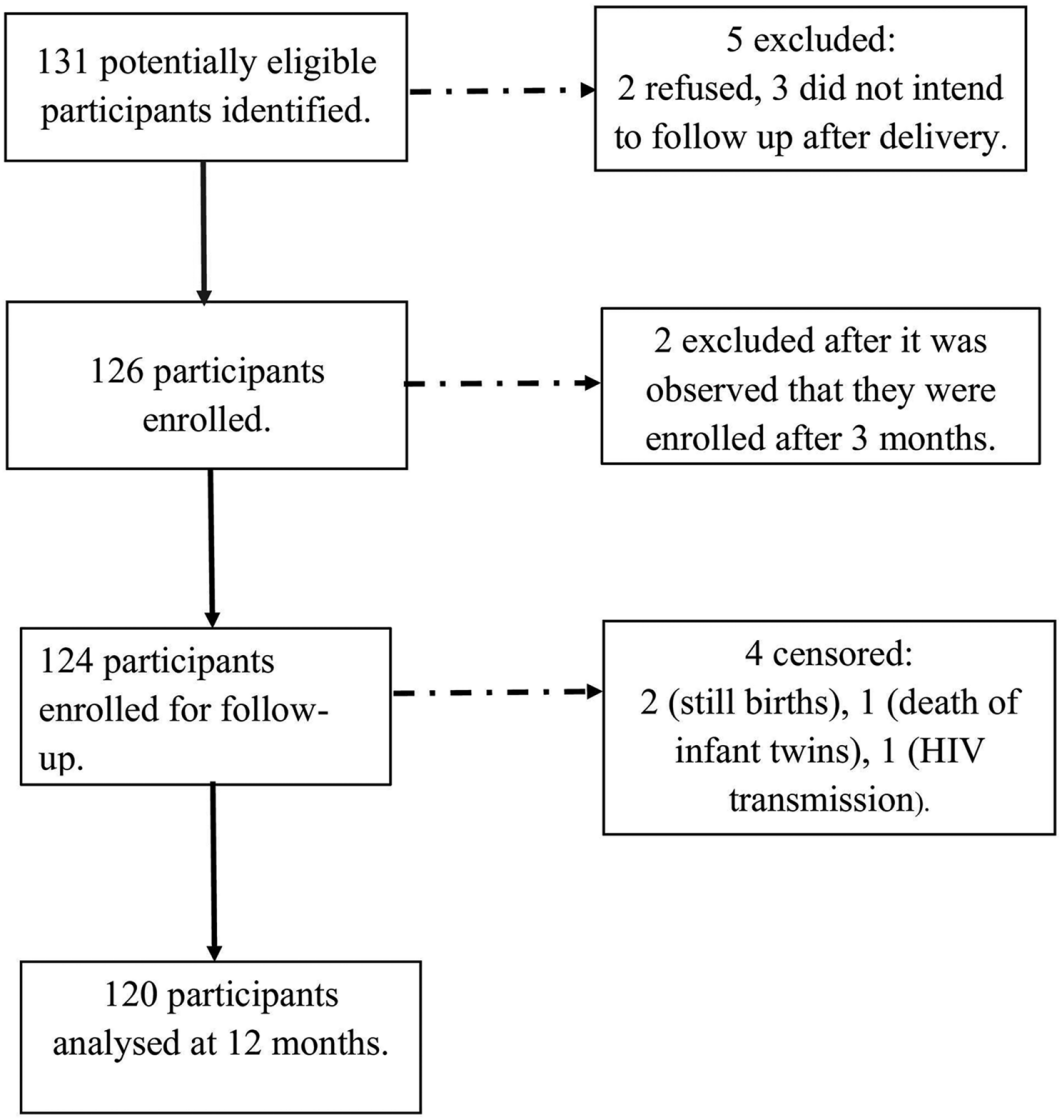
Flow diagram for study participants

We recruited 124 WLHIV into the study between 1 July 2023 and 31 December 2024. Among these, two participants experienced stillbirths, another experienced the death of her infant twins, and two cases of VT were recorded. We discontinued follow-up for four of these five participants. One participant delivered twins, one of whom was HIV-infected and the other was uninfected. We continued follow-up for this mother-infant pair with the uninfected twin. Thus, 120 mother-infant pairs were included in our final analysis (Fig. 1).

### Participant characteristics and factors associated with 12-month VL

Table 1 presents maternal baseline characteristics stratified by 12-month VL category. The median maternal age was 31.0 years (28.0, 36.3), and nearly half of the participants had attained only primary-level education (58/120; 46.7%). More than half were married (66/120; 55.0%) and self-employed (75/120; 62.5%). Most of the participants were receiving TLD (111/120, 92.5%). Despite the small number of participants on alternative regimens, VS differed significantly by ART regimen, with a higher proportion of participants suppressed on TLD regimens compared with non-TLD regimens (*p* = 0.04). Age, education, socioeconomic status, travel time to the clinic, and depression did not show significant associations with VL categorisation.

**Table 1:** Baseline characteristics stratified by VL categories at 12 months post-delivery

	Undetectable VL^a^ *N*(%) 41(33.6)	LLV^b^ *N*(%) 24(20.5)	Viremic^c^ *N*(%) 7(5.7)	Missing^d^ VL *N*(%) 48(40.2)	Overall^e^ *N*(%) 120(100.0)	*p*-value^f^
**Age (Median (Q1, Q3))**	30.0 (26.0, 36.0)	32.5 (28.0, 38.0)	30.0 (27.0, 33.5)	32.5 (29.0, 36.3)	0.25	31.0 (28.0, 36.3)
**Education Level**						0.25
None	4 (9.8%)	1 (4.2%)	2 (28.6%)	2 (4.2%)	9 (7.5%)	
Primary	15 (36.6%)	10 (41.7%)	2 (28.6%)	29 (60.4%)	58 (46.7%)	
Secondary	14 (34.1%)	8 (33.3%)	3(42.9%)	13 (27.1%)	38 (31.7%)	
Tertiary	8 (19.5%)	5 (20.8%)	0 (0.0%)	4 (8.3%)	17 (14.2%)	
**Marital Status**						> 0.90
Single	11 (26.8%)	7 (29.2%)	2 (28.6%)	14 (29.2%)	34 (28.3%)	
Married	22 (53.7%)	14 (58.3%)	3 (42.9%)	27 (56.3%)	66 (55.0%)	
Cohabiting	8 (19.5%)	3 (12.5%)	2 (28.6%)	7 (14.6%)	20 (16.7%)	
**Employment Status**						0.80
Government Employee	2 (4.9%)	0 (0%)	0 (0.0%)	2 (4.2%)	(3.3%)	
Private Employee	10 (24.4%)	7 (29.2%)	1 (14.3%)	6 (12.5%)	24 (20.0%)	
Self-employed	24(58.5%)	13 (54.2%)	5 (71.4%)	34 (68.8%)	75 (62.5%)	
Unemployed	5 (12.2%)	4 (16.7%)	1 (14.3%)	7 (14.6%)	17 (14.2%)	
**Monthly Income**						0.70
^ g^GHC < 500	18 (43.9%)	8 (33.3%)	4 (57.1%)	24 (50.0%)	54 (45.0%)	
GHC 500–1000	15 (36.6%)	11 (45.8%)	3 (42.9%)	16 (33.3%)	45 (37.5%)	
GHC > 1000–5000	8 (19.5%)	4 (16.7%)	0 (0.0%)	8 (16.7%)	20 (16.7%)	
GHC > 5000	0 (0.0%)	1 (4.2%)	0 (0.0%)	0 (0.0%)	1 (0.8%)	
**Travel Time to Hospital**						0.40
< 15 min	2 (4.9%)	2 (8.3%)	0 (0.0%)	5 (10.4%)	9 (7.5%)	
15–60 min	26 (63.4%)	14 (58.3%)	2 (28.6%)	31 (64.6%)	73 (60.8%)	
> 1–6 h	13 (31.7%)	8 (33.3%)	5 (71.4%)	12 (25.0%)	38 (31.7%)	
**Travel Distance to Hospital in kilometres (Median (Q1**,** Q3))**	7 (5, 11)	6 (4.8, 10)	12 (6, 21)	7.5 (4, 13)	7 (5, 13)	0.64
**Cost of Travel to Hospital in GHC (Median (Q1**,** Q3))**	20 (15, 45)	30 (20, 60)	30 (25, 50)	20 (19, 42.5)	20 (16, 50)	0.48
**Number of Children at Home (Median (Q1**,** Q3))**	1 (1, 2)	2 (1, 2.3)	1 (1, 2)	2 (1, 3)	1 (1, 3)	0.60
**Time of HIV Diagnosis**						0.10
During Pregnancy	0 (0.0%)	0 (0.0%)	1 (14.3%)	2 (4.2%)	3 (2.5%)	
Prior to Pregnancy	41 (100%)	24 (100%)	6 (85.7%)	46 (95.8%)	117 (97.5%)	
**VL in 3rd Trimester**						0.08
Undetectable	13 (31.7%)	4 (16.7%)	1 (14.3%)	16 (33.3%)	34 (28.3%)	
LLV	3 (7.3%)	1 (4.2%)	2 (28.6%)	11 (22.9%)	17 (14.2%)	
Viremic	1 (2.4%)	2 (8.3%)	1 (14.3%)	7 (14.6%)	11 (9.2%)	
Unknown^h^	24 (58.5%)	17 (70.8%)	3 (42.9%)	14 (29.2%)	58 (48.3%)	
**Started Current ART Regimen**						> 0.90
Pre-Pregnancy	22 (53.7%)	14 (58.3%)	4 (57.1%)	25 (52.1%)	65 (54.2%)	
During Pregnancy	17 (41.5%)	9 (37.5%)	3 (42.9%)	21 (43.8%)	50 (41.7%)	
Post-delivery	2 (4.9%)	1 (4.2%)	0 (0.0%)	2 (4.2%)	5 (4.2%)	
**Self-reported ART Adherence at Entry (Median (Q1**,** Q3))**	99.97 (85.25, 99.97)	99.97 (84.25, 99.97)	92.19 (83.58, 99.97)	99.97 (84.41, 99.97)	99.97 (84.41, 99.97)	0.66
**Depression Score at 6 Weeks Post-delivery** **(Median (Q1**,** Q3))**	3 (0, 5)	2.5 (0.3, 7.8)	4 (1, 4.5)	2 (1, 10)	3 (0, 8)	> 0.90
Missing	6	2	0	19	27	
**Number of Pregnancies** **(Median (Q1**,** Q3))**	2 (1, 3)	2 (2, 4)	1 (1, 2.5)	2.5 (1, 4)	2 (1, 4)	0.27
**Number of Live Births** **(Median (Q1**,** Q3))**	1 (1, 2)	2 (1, 2)	1.5 (1, 2)	2 (1, 3)	2 (1, 3)	0.85
**Number of Miscarriages (Median (Q1**,** Q3))**	0 (0, 1)	1 (0, 1)	0 (0, 0)	0 (0, 1.8)	0 (0, 1)	0.38
**Number of Stillbirths** **(Median (Q1**,** Q3))**	0 (0, 0)	0 (0, 0)	0 (0, 0)	0 (0, 0)	0 (0, 0)	0.79
**Maternal ART Regimen** ^**i**^						**0.04**
TL + Lop/r	0 (0%)	0 (0%)	0 (0.0%)	1 (2.1%)	1 (0.8%)	
TLD	41 (100%)	24 (100%)	6 (85.7%)	40 (83.3%)	111 (92.5%)	
TLE	0 (0%)	0 (0%)	1 (14.3%)	6 (12.5%)	7 (5.8%)	
ZDV + LD	0 (0%)	0 (0%)	0 (0.0%)	1 (2.1%)	1 (0.8%)	
**Disclosed HIV Status to Member of Household**						0.20
Yes	30 (73.2%)	18 (75.0%)	3 (42.9%)	28 (58.3%)	79 (65.8%)	
No	11 (26.8%)	6 (25.0%)	4 (57.1%)	20 (41.7%)	41 (34.2%)	
**Enrollment Site**						0.40
KBTH	27 (65.9%)	16 (66.7%)	6 (85.7%)	26 (54.2%)	75 (62.5%)	
GARH	14 (34.1%)	8 (33.3%)	1 (14.3%)	22 (45.8%)	45 (37.5%)	

^a^Undetectable viral load (VL) was defined as < 20 copies/ml^b^Low-level viremia (LLV) was defined as a VL between 20 and 1000 copies/ml^c^Viremia was defined as VL > 1000 copies/ml.^d^Missing VL is the absence of a documented maternal VL result at 12 months post-delivery for participants who could not be reached by phone and remained untraceable in the national database for more than 3 months after their scheduled appointment.^e^Total excludes individuals who were censored from the 12-month analysis (2 due to stillbirth, 1 due to early infants death, and 1 case of VT).^f^Chi-square analysis or Fisher’s test for categorical variables, ANOVA or Kruskal–Wallis tests, depending on data normality for continuous variables. Statistical significance was set at 2-sided *p* ≤ 0.05.^g^Ghana’s currency- The Ghana Cedi^h^Includes 57 women who were enrolled post-delivery, and 1 who was enrolled during the 3rd trimester but who did not have a VL available^i^Maternal ART Regimen - TL + Lop/r =tenofovir, lamivudine, lopinavir/ritonavir; TLD= tenofovir, lamivudine, dolutegravir; TLE = tenofovir, lamivudine, efavirenz; ZDV + LD = zidovudine, lamivudine, dolutegravir

### VL suppression and undetectable VL at 12 months post-delivery

At 12 months post-delivery, 65/120 (54.2%) participants achieved VS (≤ 1000 copies/mL), and 41/120 (34.2%) had undetectable VLs (< 20 copies/mL). Seven (5.8%) participants had confirmed VLs greater than 1000 copies/mL.

### Trend of VL categories

**Fig. 2:**
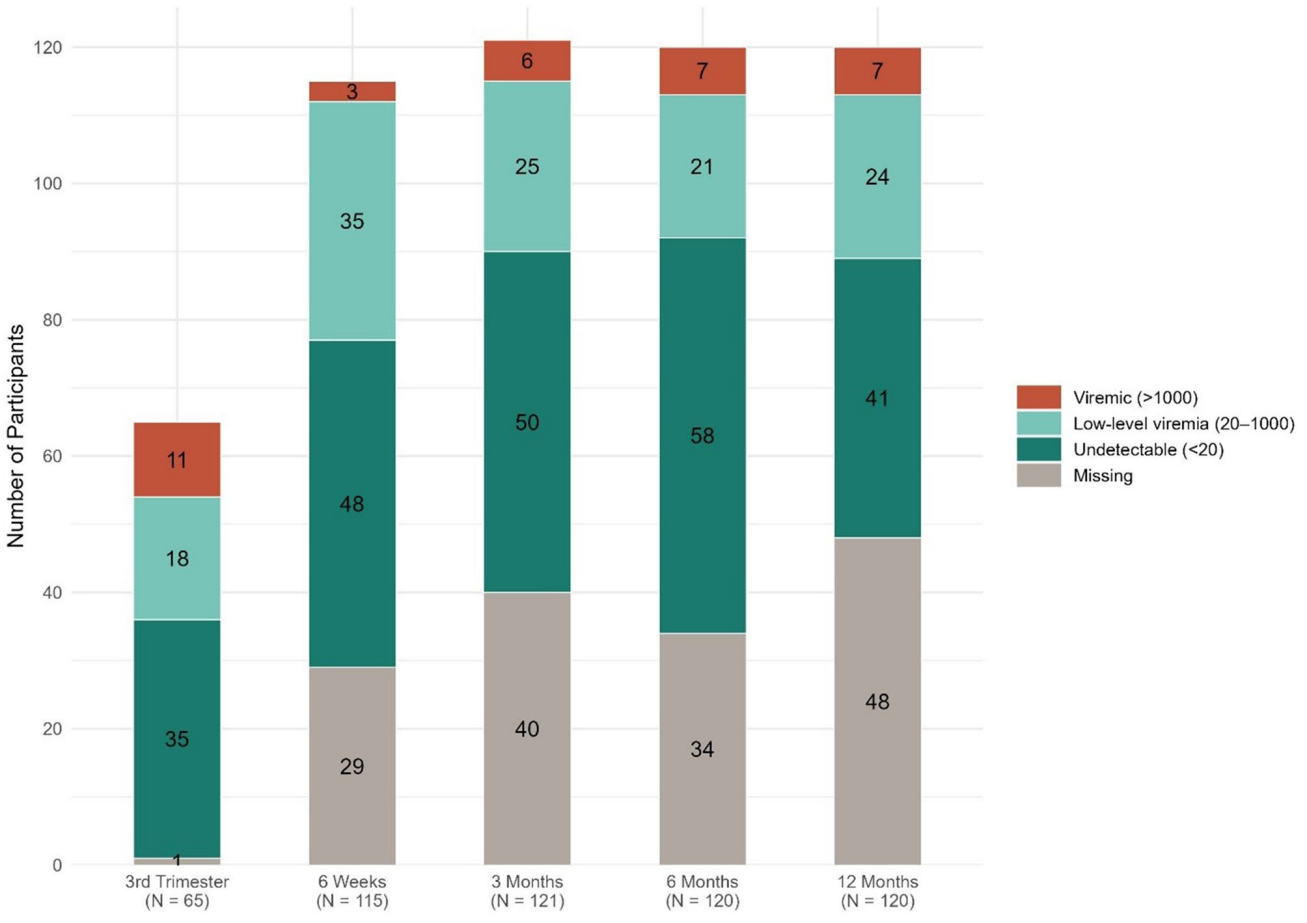
VL during pregnancy and post-delivery

A total of 48/120 (40.0%) participants were LTFU and therefore had no VL data available at 12 months. The overall VS (documented ≤ 1000 copies/ml) decreased from 81.5% (53/65) in the third trimester to 54.2% (65/120) by 12 months post-delivery. The proportion of participants with documented high-level viremia (> 1000 copies/mL) remained low and relatively stable (approximately 5–11%). However, missing data increased substantially over time, from 1.5% at entry to 40.0% at 12 months. Most individuals with missing data did not return for care at the study sites and were unreachable by our study team. These trends are summarised in Table 2, Fig. 2.

**Table 2:** VL categories and the corresponding proportions of women by VL values at each study visit

VL category	Third trimesterN (%)	6 weeksN (%)	3 months N (%)	6 months N (%)	12 months N (%)
Total	65 (100)	115 (100)	121 (100)	120 (100)	120 (100)
Undetectable	35 (53.9)	48 (41.7)	50 (41.3)	58 (48.3)	41 (34.2)
Low-level Viremia	18 (27.7)	35 (30.4)	25 (20.7)	21 (17.5)	24 (20.0)
Viremia	11 (16.9)	3 (2.6)	6 (5.0)	7 (5.8)	7 (5.8)
Missing	1 (1.5)	29 (25.2)	40 (33.1)	34 (28.3)	48 (40.0)
Censored^a^	59	9	3	4	4

^a^The 59 women censored at the third trimester enrolled post-delivery, whilst the 9 women censored from the 6-week analysis were enrolled after their 6-week postpartum date. At 3 months, 2 women were censored due to stillbirth, and 1 due to early infant death. At 6 months, the same participants were censored due to stillbirth (2), early infant death (1), and 1 new case of VT. Those same 4 individuals continued to be censored at 12 months. Percentages are calculated using the total number of non-censored participants

### Time-to-event analysis

**Fig. 3:**
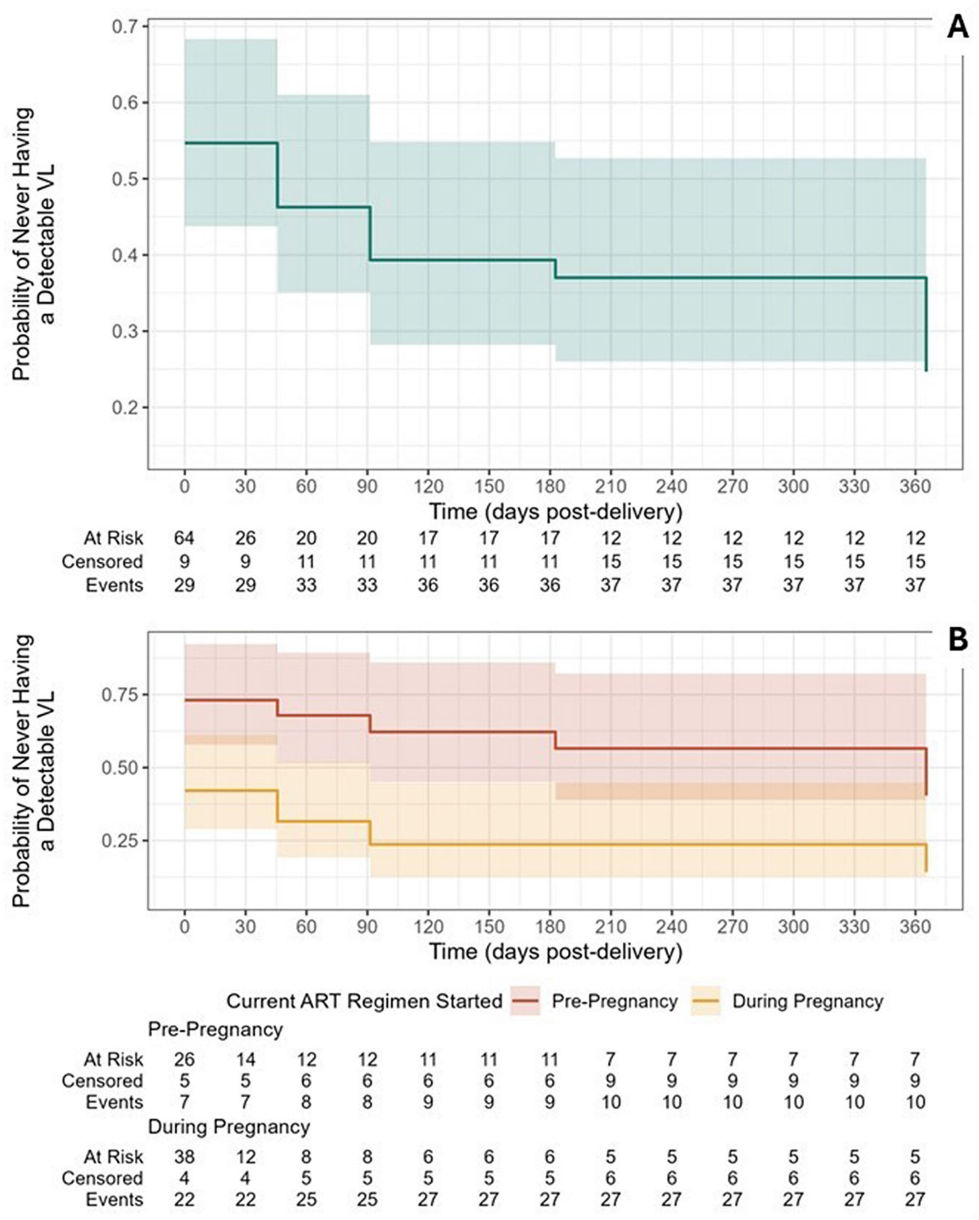
Kaplan-Meier curves of time to failure (detectable viral load). The Kaplan-Meier curves show the probability of never having a measured detectable VL over the course of the study period. Panel A shows the overall curve for all individuals enrolled during the 3rd trimester; Panel B shows the curves stratified by timing of initiation of current ART regimen (pre-pregnancy versus during pregnancy)

Among the 64 individuals with a VL value during the third trimester, 41 had a detectable VL within 12 months. Individuals who started their current ART regimen during pregnancy had a significantly higher hazard of having a detectable VL in the year post-delivery than individuals who started their current regimen pre-pregnancy (HR 2.44 [1.24, 4.80]). Kaplan-Meier curves overall and stratified by timing of current ART regimen are presented in Fig. 3. Both curves begin with a probability less than 1.0 because a substantial proportion of women had detectable VL at the measurement closest to the infant’s birth.

### Lost to follow up

Table 3 presents a more detailed look at the similarities and differences between those who were LTFU and those who remained in care at 12 months post-delivery. Among the measured variables, only the ART regimen and VL in the third trimester were associated with LTFU. Among the 48 infants whose mothers were lost to follow-up, 36 (75.0%) lacked a documented 9-month PCR result, resulting in unknown HIV serostatus. Feeding information was available for 12 of these infants at 3 months or later, all of whom were breastfed or mixed-fed.

**Table 3:** Baseline characteristics categorised by whether VLs were available at 12 months (no VL=lost to follow-up), excluding individuals who were censored

Maternal demographic and clinical baseline characteristics	Not lost to follow-up, *N* = 72	Lost to follow-up, *N* = 48	Overall *N* = 120	*P* value
**Maternal Age (Median (Q1**,** Q3))**	30.0 (26.8, 36.3)	32.5 (29.0, 36.3)	31.0 (28.0, 36.3)	0.32
**Maternal Education Level**				0.08
None	7 (9.7%)	2 (4.2%)	9 (7.5%)	
Basic	27 (37.5%)	29 (60.4%)	56 (46.7%)	
Secondary	25 (34.7%)	13 (27.1%)	38 (31.7%)	
Tertiary	13 (18.1%)	4 (8.3%)	17 (14.2%)	
**Marital Status**				0.9
Single	20 (27.8%)	14 (29.2%)	34 (28.3%)	
Married	39 (54.2%)	27 (56.3%)	66 (55.0%)	
Cohabiting	13 (18.1%)	7 (14.6%)	20 (16.7%)	
**Maternal Employment Status**				0.4
Government Employee	2 (2.8%)	2 (4.2%)	4 (3.3%)	
Private Employee	18 (25.0%)	6 (12.5%)	24 (20.0%)	
Self-employed	42 (58.3%)	33 (68.8%)	75 (62.5%)	
Unemployed	10 (13.9%)	7 (14.6%)	17 (14.2%)	
**Monthly Income**				0.8
GH < 500	30 (41.7%)	24 (50.0%)	54 (45.0%)	
GH 500–1000	29 (40.3%)	16 (33.3%)	45 (37.5%)	
GH > 1000–5000	12 (16.7%)	8 (16.7%)	20 (16.7%)	
GH > 5000	1 (1.4%)	0 (0.0%)	1 (0.8%)	
**Time to Travel to Hospital**				0.3
< 15 min	4 (5.6%)	5 (10.4%)	9 (7.5%)	
15–60 min	42 (58.3%)	31 (64.6%)	73 (60.8%)	
1–6 h	26 (35.1%)	12 (25.0%)	38 (31.7%)	
**Distance to Travel to Hospital (km) (Median (Q1**,** Q3))**	7 (5, 12)	7.5 (4, 13)	7 (5, 13)	0.82
**Cost to Travel to Hospital GHC Median (Q1**,** Q3))**	30 (16, 50)	20 (19, 42.5)	20 (16, 50)	0.65
**Number of Children at Home (Median (Q1**,** Q3))**	1 (1, 2)	2 (1, 3)	1 (1, 3)	0.26
**Diagnosis Timing**				0.6
During Pregnancy	1 (1.4%)	2 (4.2%)	3 (2.5%)	
Prior to Pregnancy	71 (98.6%)	46 (95.8%)	117 (97.5%)	
**VL in 3rd Trimester**				**0.003**
Undetectable	18 (25.0%)	16 (33.3%)	34 (28.3%)	
LLV	6 (8.3%)	11 (22.9%)	17 (14.2%)	
Viremic	4 (5.6%)	7 (14.6%)	11 (9.2%)	
Unknown	44 (61.1%)	44 (61.1%)	58 (48.3%)	
**Started Current ART Regimen**				0.9
Pre-Pregnancy	40 (55.6%)	25 (52.1%)	65 (54.2%)	
During Pregnancy	29 (40.3%)	21 (43.8%)	50 (41.7%)	
Post-delivery	3 (4.2%)	2 (4.2%)	5 (4.2%)	
**Self-reported ART Adherence at Entry (Median (Q1**,** Q3))**	99.97 (84.41, 99.97)	99.97 (84.41, 99.97)	99.97 (84.41, 99.97)	0.54
**Depression Score at 6 Weeks Post-partum (Median (Q1**,** Q3))**	3 (0, 5)	2 (1, 10)	3 (0, 8)	0.47
Missing	9	20	29	
**Number of Pregnancies (Median (Q1**,** Q3))**	2 (1, 3)	2.5 (1, 4)	2 (1, 4)	0.15
**Number of Live Births (Median (Q1**,** Q3))**	1 (1, 2)	2 (1, 3)	2 (1, 3)	0.42
**Number of Miscarriages (Median (Q1**,** Q3))**	0 (0, 1)	0 (0, 1.8)	0 (0, 1)	0.86
**Number of Stillbirths (Median (Q1**,** Q3))**	0 (0, 0)	0 (0, 0)	0 (0, 0)	0.83
**Maternal ART Regimen**				**0.003**
Tenofovir, lamivudine, lopinavir	0 (0%)	1 (2.1%)	1 (0.8%)	
Tenofovir, lamivudine, dolutegravir	71 (98.6%)	40 (83.3%)	111 (92.5%)	
Tenofovir, lamivudine, efavirenz	1 (1.4%)	6 (12.5%)	7 (5.8%)	
Zidovudine, lamivudine, dolutegravir	0 (0%)	1 (2.1%)	1 (0.8%)	
**Disclosed HIV Status to Member of Household**				0.2
Yes	51 (70.8%)	28 (58.3%)	79 (65.8%)	
No	21 (29.2%)	20 (41.7%)	41 (34.2%)	
**Enrollment Site**				0.12
Korle Bu Teaching Hospital	49 (68.1%)	26 (54.2%)	75 (62.5%)	
Ridge Regional Hospital	23 (31.9%)	22 (45.8%)	45 (37.5%)	

### Infant outcomes

Among the 124 pregnant women enrolled, two stillbirths and the death of infant twins occurred at 6 weeks. The twins were found unresponsive when their mother attempted to wake them from sleep. Two cases of VT of HIV were identified—one at six weeks and another at six months. The maternal VLs closest to the time of transmission were 669 copies/mL and 64 copies/mL, respectively (Table 4). All enrolled infants received antiretroviral (ARV) prophylaxis from birth as per the national policy. The infant who tested positive at six months had a negative DNA PCR result at six weeks. HIV infection was confirmed following repeat testing prompted by the presentation of otitis media and a generalised rash at 5.5 months.

**Table 4:** Maternal characteristics of infants with vertical transmission of HIV

Maternal characteristics of infants with vertical transmission of HIV	Infant 1	Infant 2
Maternal ART regimen	Tenofovir, lamivudine, dolutegravir	Tenofovir, lamivudine, dolutegravir
Maternal VL at study entry copies/ml	Undetectable	669
Maternal VL at 6 weeks/copies/ml	Undetectable	669^1^
Maternal VL at 3 months copies/ml	Undetectable	649
Maternal VL at 6 months copies/ml	64	249
Type of pregnancy	Singleton	1st twin
Type of feeding 6 weeks	EBF^2^	EBF
Type of feeding 3 months	EBF	EF^3^
Type of feeding at 6 months	EBF	EF
VT report	6 months	6 weeks

^1^Mother entered the study at 6 weeks, ^2^EBF – Exclusive breastfeeding, ^3^EF- Exclusive formula, Maternal monitoring was stopped following documented infant infection in singletons but continued in the mother of infant 2 due to the presence of an uninfected twin

## Discussion

### Viral load outcomes

We conducted this prospective cohort study to understand how well VS is maintained post-delivery in WLHIV in Ghana, most of whom were breastfeeding throughout the monitoring period, consistent with the practices of the general population. Despite initial high rates of VS 81.5% at study entry, the percentage with confirmed VS declined to 54.2% at 12 months, with a high rate of LTFU, 48 (40.0%). The decrease in confirmed versus likely cases of VS may reflect challenges in retention in care, adherence fatigue, and functional gaps in post-delivery care [18]. In a South African cohort of WLHIV, the proportion with VS decreased from 84% at six weeks postpartum to 67% at 12 months post-delivery [19]. These findings highlight a critical vulnerability during the breastfeeding period—a key window for VT. Despite lifelong dolutegravir-based regimens, sustaining virologic control beyond pregnancy requires intensified support—an ongoing concern in sub-Saharan Africa (SSA) where the incidence of post-delivery VS is often lower than that during pregnancy [10, 12]. Maternal VL is a key determinant of VT risk [20]. In this study cohort, we provided nevirapine prophylaxis for infants whose mothers were breastfeeding and whose VL was > 1000 copies/ml, as recommended in the South African national guidelines for prevention of HIV VT [21]. However, two transmissions occurred from mothers with detectable VLs, but never measured to be > 1000 copies/mL. These findings reinforce that VL < 1000 copies/mL can still pose a transmission risk via breast milk, which is consistent with reports in the literature [22, 20]. While the transmission risk associated with LLV (50–1000 copies/mL) remains unclear, evidence suggests that even low levels of viremia can contribute to viral shedding in breast milk and increase the risk of VT [20, 23]. In our study, the rate of LLV was substantial, ranging from 17.4 to 30.4% in the year post-delivery, and the true risk of viremia was likely underestimated, given the unknown virological status of those who were LTFU. This underscores the need for more frequent VL testing; ongoing engagement with WLHIV throughout the year after delivery and the period of breastfeeding, if longer than a year; and consideration of tailored infant prophylaxis based on maternal VL status. Notably, none of the mothers with VL > 1000 copies/mL in our study transmitted HIV through breastfeeding, which could have been due both to the small number of women in this group and to the provision of additional infant prophylaxis when maternal viremia reached this threshold. Given that exclusive breastfeeding remains critical in low-resource settings, VL suppression must be sustained throughout the breastfeeding period. A systematic review and meta-analysis of 147 studies involving over 82,000 mother–child pairs assessed the risk of HIV vertical transmission by maternal VL. Perinatal transmission risk was 0.2% with VL < 50 copies/mL, 1.3% with 50–999 copies/mL, and 5.1% with ≥ 1000 copies/mL. Notably, no transmissions occurred among women who conceived on ART and maintained an undetectable VL at delivery. However, data on transmission during breastfeeding were limited, and although transmission was rare, it was not zero among women with suppressed VLs [24]. The findings support the U = U principle during pregnancy and delivery; however, the safe VL threshold during breastfeeding remains uncertain, with only undetectable VLs appearing to provide reliable protection. In our study, the likelihood of never having experienced a detectable VL was below 40% at six months after delivery.This is particularly concerning in the context of WHO recommendations for exclusive breastfeeding for the first six months of life and the uncertainty regarding the transmission risk associated with LLV. Studies have shown that infant prophylaxis is effective at preventing transmission during breastfeeding [25], even when the mother has a detectable VL. In settings with low rates of post-delivery VS, universal prophylaxis for breastfeeding infants may therefore be an ideal strategy to limit transmission. Cost-effectiveness studies on this topic are limited, but one study from South Africa found that increasing nevirapine coverage was cost-effective and improved HIV-free survival [26]. Our data suggests that in settings where universal prophylaxis for all breastfeeding infants with mothers living with HIV is infeasible, risk stratification by timing of maternal ART regimen, may help with prioritization of resources. Future studies evaluating additional factors associated with risk of maternal viral non-suppression may help to further elucidate opportunities to mitigate risk.

### Factors associated with VL outcomes

All women with undetectable VL were on TLD. Dolutegravir’s high potency and resistance barrier make it the preferred first-line ART [6]. In our study, factors such as age, education, socioeconomic status, travel time, and depression showed no significant associations with our outcomes of interest. In Malawi, detectable VL in the early post-delivery period signalled an increased risk of ongoing viremia at both 12 and 24 months post-delivery, whereas suboptimal adherence was significantly associated with loss of VS among those who had VS at enrollment and those with persistently detectable VL [18], findings that were not replicated in our study.

### Data gaps and loss to follow-up

A key limitation of this study was the increasing proportion of missing maternal VL data over time, particularly at 12 months post-delivery, when 40.0% of VL results were unavailable due to LTFU. This reflects systemic barriers, such as patient disengagement from care, inadequate appointment tracking, or logistical issues related to travel. This high rate of LTFU is particularly noteworthy, given that participants were reimbursed for travel costs in an effort to reduce the burden to new mothers. In a real-world setting, where transportation costs must be borne by patients and are a significant barrier to engagement in care [27], rates of LTFU therefore have the potential to be even higher than were found in this study. In Ghana, a prior qualitative study revealed that retention in HIV care was more difficult post-delivery than during pregnancy, due to poor maternal health, sociocultural norms around childbirth, and economic challenges such as unemployment and debt [9]. These gaps in post-delivery VL monitoring are concerning, given the known risk of transmission during breastfeeding and the dynamic nature of adherence during this period. The inability to ascertain VS status in a substantial proportion of women limits the timely identification of those at risk of transmitting HIV to their infants. In our study population, most infants whose mothers were lost to follow-up did not have an HIV PCR result available beyond 6 weeks of life and may have continued to receive breastmilk. For these infants, the risk of VT remains unknown. In the absence of either ongoing maternal or infant VL monitoring, these infants may be at risk of delayed HIV diagnosis. This underscores the urgent need for more robust post-delivery care models, including point-of-care VL testing, improved follow-up systems, and differentiated care approaches to keep mothers engaged throughout the breastfeeding period. Strengthening these systems could reduce data gaps, ensure more accurate VT risk stratification, and enable targeted infant prophylaxis and support for maternal adherence.

### Clinical and programmatic implications

To minimise VT risk, maternal VL should be monitored more frequently during breastfeeding, with prompt actions taken for non-suppression. Interventions such as community-based adherence groups, routine post-delivery support, and continuous counselling may mitigate suppression loss [23]. Our data suggest that scaling up TLD use is crucial for maintaining sustained virologic control and preventing VT.

### Limitations

Our study has several limitations. Although the study benefits from a longitudinal design, its limited statistical power may have hindered the identification of some risk factors. The sample size was relatively modest, and incomplete follow-up resulted in some missing data, which may reduce the precision of our estimates and limit generalizability beyond the study population. Although we implemented active follow-up and support strategies to maximise retention, some participants were lost to follow-up or had missing VL measurements. Although HIV transmission occurred in two infants at relatively low measured viral loads (64 and 669 copies/mL), the absence of more frequent (e.g., monthly or continuous) viral load assessments limits our ability to determine whether these values reflected sustained low-level viremia or transient viral blips. Consequently, we cannot exclude the possibility that unmeasured intermittent increases in viral load contributed to transmission. The infant outcome sample is too small for robust inference regarding transmission risks. These factors could introduce bias and should be considered when interpreting the findings.

## Conclusions

The substantial decline in post-delivery VS and two cases of infant infection amidst low maternal viremia underscore missed opportunities in the prevention of VT efforts in Ghana. Urgent action is needed to improve maternal VL monitoring during the breastfeeding period, provide infant prophylaxis when needed, and bolster post-delivery retention in care. Such measures are crucial for achieving the elimination of VT and protecting maternal and infant health in high-burden settings. Further studies are needed to determine the levels of maternal VL associated with VT of HIV during breastfeeding and to assess the effectiveness of prophylaxis for infants of mothers with varying degrees of viremia. Current evidence indicates that any detectable maternal VL may still pose a risk of transmission.

## Supplementary Information

Below is the link to the electronic supplementary material.

Supplementary Material 1

## Data Availability

The datasets used and or analysed during the current study are available from the corresponding author upon reasonable request.

## Abbreviations

AIDS: Acquired immunodeficiency syndrome
ANOVA: Analysis of variance
ARV: Antiretroviral
ART: Antiretroviral therapy
CI: Confidence interval
DNA: Deoxyribonucleic acid
EBF: Exclusive breastfeeding
EF: Exclusive formula
GARH: Greater Accra Regional Hospital
GH₵: Ghana cedi
HIV: Human immunodeficiency virus
KBTH: Korle Bu Teaching Hospital
LLV: Low-level viremia
LTFU: Lost to follow-up
PCR: Polymerase chain reaction
REDCap: Research Electronic Data Capture
SD: Standard deviation
SSA: Sub-Saharan Africa
TLD: Tenofovir, lamivudine, and dolutegravir
U = U: undetectable = untransmissible
VL: Viral load
VS: Viral suppression
VT: Vertical transmission
WLHIV: Women living with HIV
WHO: World Health Organization
