# Supplementary material for: Viral load suppression among pregnant and breastfeeding women living with HIV in Ghana: a prospective longitudinal study
Source: BMC Infect Dis. 2026 Apr 14;26:1012. doi: 10.1186/s12879-026-13282-x (PMC13200356; doi:10.1186/s12879-026-13282-x)
Supplement: Supplementary file 1 — Supplementary Material 1 [file 12879_2026_13282_MOESM1_ESM.docx]

SUPPLEMENTARY FILE 1

DATA COLLECTING TOOL

**A. MEDICAL RECORD REVIEW AT STUDY ENTRY**

Study Site: 1. Korle Bu Teaching Hospital 2. Ridge Regional Hospital

Date of medical record review ……………………………………..(day, month, year)

Time of medical record review : Before delivery After Pregnancy (circle one)

Study No……………..

1. Maternal Age --------------------------------------(years)

2. Maternal Date of HIV Diagnosis ------------------------------------(day/month/year)

3. Current HIV treatment regimen

Circle all that apply

| ARV drug |  |
| --- | --- |
| Drug | Check all that apply |
| Zidovudine |  |
| Abacavir |  |
| Tenofovir |  |
| Emtricitabine |  |
| Lamivudine |  |
| Nevirapine |  |
| Efavirenz |  |
| Dolutegravir |  |
| lopinavir/r |  |
| tenofovir/lamivudine |  |
| tenofovir/emtricitabine |  |
| lamivudine/zidovudine |  |
| abacavir/lamivudine |  |
| dolutegravir/ lamivudine/tenofovir |  |
| dolutegravir/emtiricitabine/tenofovir |  |
| abacavir/lamivudine/ efavirenz |  |
| zidovudine/lamivudine/efavirenz |  |
| tenofovir/lamivudine/ efavirenz |  |

4. Date of initiation of current treatment --------------------------------------(day/month/year)

5. i. Has patient had a viral load done before? Yes No ( if yes, go to question 6 if no, go to 5ii)

5ii. If no, what is the reason? a. New diagnosis b. Transfer to this hospital c. Never done

d. other reason (state it).............................

6. Mother’s last viral load …………………………..(copies/ml)

7. Date of last viral load...………………………… (day/month/year)

8. WHO clinical stage (Circle the correct one) a. Stage 1 b. Stage 2 c. Stage 3 d. Stage 4

9. Maternal health (This section is recorded only after delivery)

| Tick all that apply | Maternal Illness |
| --- | --- |
|  | Preeclampsia/eclampsia |
|  | Gestational diabetes |
|  | Premature rupture of membranes |
|  | Cervical Incompetence |
|  | Post-partum haemorrhage |
|  | Sickle cell disease |
|  | Malaria |
|  | Syphilis |
|  | Hepatitis B |
|  | Tuberculosis |
|  | Anaemia not requiring blood transfusion |
|  | Anaemia requiring blood transfusion |
|  | Other ......(Please state) |

10. Is the mother on other medication? Yes No

If yes, state it

**B. ENTRY VISIT QUESTIONNAIRE IN THE 3^RD^ TRIMESTER (BASELINE)**

Study Site: 1. Korle Bu Teaching Hospital 2. Ridge Regional Hospital

Date of interview……………………………………(day, date, year)

Study No:………………………………………..

1. Gestational week of pregnancy (Number of weeks pregnant) ………………………………….

2a. The number of pregnancies (the number of times the woman has been pregnant irrespective of outcome)

i. The number of births that were live………………

ii. The number of births that were still born……

iii. The number of miscarriages and their weeks of gestation

2b. Number of twins? ……………Number of other multiplies ……………(state the type e.g. triplets)

3. Maternal highest educational level. (Circle only one)

None Basic Secondary Tertiary

4. Marital status

a. Single

b. Married

c. Cohabiting

d. Divorced

e. Widowed

f. Separated

4a. The number of children in your home that you take care of? …………………..

4b. Out of this number how many did you give birth to?

5. How long does it take you to get to the hospital?

1. Less than 15 minutes
2. 15-60 minutes
3. 1-6 hours
4. More than 6 hours
5. Unknown

6. Cost of transportation to the hospital (state in Ghana cedis)………………………..

7. Mother’s Employment status

a. Government employee

b. Private employee

c. Self employed

d. Unemployed

e. Other------------------------

8. Monthly income

1. GH < 500
2. GH 500-1000
3. GH > 1000- 5000
4. GH >5000

9. Disclosure

Have you disclosed your status to anyone in your house?

Yes No

If yes, what is your relationship with that person?

1. Spouse
2. A parent
3. A child
4. A sibling
5. A friend
6. Grandparent
7. Other ( state it )

**10. 3-item self-report measure for Medication Adherence**

a. In the last 30 days, how many *days* did you miss at least one dose of any of your ARVS?

Write in the number of days: ____ (0–30)

b. In the last 30 days, how good a job did you do at taking your ARVs in the way you were supposed to?

1. Very poor
2. Poor
3. Fair
4. Good
5. Very good
6. Excellent

c. In the last 30 days, how often did you take your ARV in the way you were supposed to?

1. □ Never
2. □ Rarely
3. □ Sometimes
4. □ Usually
5. □ Almost always
6. □ Always

**C. ENTRY VISIT QUESTIONNAIRE AFTER DELIVERY**

Study Site: 1. Korle Bu Teaching Hospital 2. Ridge Regional Hospital

Date of interview……………………………………( day, month, year)

Study No:

1. Postpartum period (Number of weeks after delivery) ………………………………….

2a. Maternal parity ……………………………………( the number of times she has given birth irrespective of the outcome)

2b. The number of live births……………… The number of still births…………………

3. Maternal highest educational level. (Circle only one)

None Basic Secondary Tertiary

4. Marital status

a. Single

b. Married

c. Cohabiting

d. Divorced

e. Widowed

f. Separated

5a. The number of children in your home that you take care of ?…………………..

5b. Out of this number how many are yours?

6. How long does it take you to get to the hospital

1. Less than15 minutes
2. 15-60 minutes
3. 1-6 hours
4. More than 6 hours
5. Unknown

7. Cost of transportation to the hospital

8. Mother’s Employment status

a. Government employee

b. Private employee

c. Self employed

d. Unemployed

e. Other------------------------

9. Monthly Income

1. GH < 500
2. GH 500-1000
3. GH > 1000- 5000
4. GH >5000

10. Pregnancy outcomes

| **Tick all that apply** | **Pregnancy Outcomes** |
| --- | --- |
|  | Live |
|  | Stillbirth |
|  | Prematurity |
|  | Low 5 min Apgar score (Apgar score < 7 at 5 mins) |
|  | Small for age |

11. Type of Infant feeding

Exclusive breastfeeding Formula Feeding Mixed Feeding

Other-----------------(state it)

12. Maternal health during breastfeeding (skip if exclusively formula feeding)

(Circle only one)

Normal Cracked nipples Mastitis

13 i. If cracked nipples or mastitis, was there bleeding? Yes No

14 ii. If yes, how long did bleeding last?

a. < 1 day

b. 1 day

c. 2-4 days

d. 4-6 days

e. >6 days

iii. Did cracked nipples or mastitis require treatment? Yes No

iv. If yes, type of treatment

a. Antibiotics

b. Incision and drainage

c. Other

15. Have you disclosed your status to anyone in your house?

Yes No

If yes, what is your relationship with that person?

1. Spouse
2. A parent
3. A child
4. A sibling
5. A friend
6. Grandparent
7. Other ( state it )

**16. 3-item self-report measure for Medication Adherence**

a. In the last 30 days, how many *days* did you miss at least one dose of any of your ARVS?

Write in the number of days: ____ (0–30)

b. In the last 30 days, how good a job did you do at taking your ARVs in the way you were supposed to?

1. Very poor
2. Poor
3. Fair
4. Good
5. Very good
6. Excellent

c. In the last 30 days, how often did you take your ARV in the way you were supposed to?

1. Never
2. Rarely
3. Sometimes
4. Usually
5. Almost always
6. Always

**D. 6-WEEK VISIT**

Study Site: 1. Korle Bu Teaching Hospital 2. Ridge Regional Hospital

Date of interview………………………………. (date/month/year)

Study No:

1. Postpartum period (Number of weeks after delivery) ………………………………….

2. Type of Infant feeding (circle one)

a. Exclusive breastfeeding

b. Formula Feeding only

c. Mixed Feeding

d. Other………….. (State)

3. Maternal health during breastfeeding (skip to 4 if formula feeding)

(circle one) Normal Cracked nipples Mastitis

3i. If cracked nipples or mastitis, was there bleeding? Yes No

If yes, how long did bleeding last?

a. < 1 day

b. 1 day

c. 2-4 days

d. 4-6 days

e. >6 days

ii. Did cracked nipples or mastitis require treatment? Yes No

iii. If yes, type of treatment

a. Antibiotics

b. Incision and drainage

c. Other

4. Since your last study visit, have you newly disclosed your status?

Yes No

If yes, what is your relationship with that person?

1. Spouse
2. A parent
3. A child
4. A sibling
5. A friend
6. Grandparent
7. Other (state it**)......**

**5. 3-item self-report measure for Medication Adherence**

a. In the last 30 days, how many *days* did you miss at least one dose of any of your ARVS?

Write in the number of days: ____ (0–30)

b. In the last 30 days, how good a job did you do at taking your ARVs in the way you were supposed to?

1. Very poor
2. Poor
3. Fair
4. Good
5. Very good
6. Excellent

c. In the last 30 days, how often did you take your ARV in the way you were supposed to?

1. Never
2. Rarely
3. Sometimes
4. Usually
5. Almost always
6. Always

**Data extraction form at 6 weeks**

1. Mother’s viral load result at 6 weeks……………………………………….(copies/ml).

2. Infant’s current health status (circle one)

i. Alive and well Alive but unwell Dead

ii. If unwell, what is the diagnosis?

1. Pneumonia
2. TB
3. Gastroenteritis
4. Sepsis
5. Malaria
6. Prolonged jaundice
7. Other (please state)
8. Unknown

iii. Has there been any interval illness? (Illness since birth and this visit)

Yes No

iv.If yes, what was the diagnosis?

1. Pneumonia
2. TB
3. Gastroenteritis
4. Sepsis
5. Malaria
6. Prolonged jaundice
7. Other (please state)
8. Unknown

v. If dead, record the cause of death from the infant’s medical records

3. Infant’s PCR results at 6 weeks……………..

**E. 3-MONTH VISIT**

Study Site: 1. Korle Bu Teaching Hospital 2. Ridge Regional Hospital

Date of interview…………………………(day, date, year)

Study No:………………………………….

1. Postpartum period (Number of weeks after delivery) ………………………………….

2. Type of Infant feeding (circle one)

Exclusive breastfeeding Formula Feeding Mixed Feeding

3.i. If cracked nipples or mastitis, was there bleeding? Yes No

If yes, how long did bleeding last?

a. < 1 day

b. 1 day

c. 2-4 days

d. 4-6 days

e. >6 days

ii. Did cracked nipples or mastitis require treatment? Yes No

iii. If yes, type of treatment

a. Antibiotics

b. Incision and drainage

c. Other

Disclosure (for those who have not disclosed yet)

4. Since your last study visit, have you newly disclosed your status?

Yes No

If yes, what is your relationship with that person?

1. Spouse
2. A parent
3. A child
4. A sibling
5. A friend
6. Grandparent
7. Other (state it**)......**

**5. 3-item self-report measure for Medication Adherence**

a. In the last 30 days, how many *days* did you miss at least one dose of any of your ARVS?

Write in the number of days: ____ (0–30)

b. In the last 30 days, how good a job did you do at taking your ARVs in the way you were supposed to?

1. Very poor
2. Poor
3. Fair
4. Good
5. Very good
6. Excellent

c. In the last 30 days, how often did you take your ARV in the way you were supposed to?

1. Never
2. Rarely
3. Sometimes
4. Usually
5. Almost always
6. Always

**Data extraction form at 3 months**

1. Mother’s viral load result at 3 months……………………………………….(copies/ml).

2. Infant’s current health status (circle one)

i. Alive and well Alive but unwell Dead

ii. If unwell now, what is the diagnosis?

1. Pneumonia
2. TB
3. Gastroenteritis
4. Sepsis
5. Malaria
6. Prolonged jaundice
7. Other (please state)
8. Unknown

iii. Has there been any interval illness? (Illness since last seen and this visit)

Yes No

iv. If yes, what was the diagnosis?

a. Pneumonia

b. TB

c. Gastroenteritis

d. Sepsis

e. Malaria

f. Prolonged jaundice

g. Other (please state)

h. Unknown

v. If dead, record the cause of death from the infant’s medical records

**F. 6-MONTH VISIT**

Study Site: 1. Korle Bu Teaching Hospital 2. Ridge Regional Hospital

Date of interview

Study No:

1. Postpartum period (Number of weeks after delivery) ………………………………….

2. Type of Infant feeding (circle one)

1. Exclusive breastfeeding
2. Exclusive formula feeding
3. Complementary feeds without breastmilk or infant formula
4. Complementary feeding + breastmilk
5. Complementary feeds + formula
6. Complementary feeds + formula + breastmilk
7. Other

3i. If cracked nipples or mastitis, was there bleeding? Yes No

If yes, how long did bleeding last?

a. < 1 day

b. 1 day

c. 2-4 days

d. 4-6 days

e. >6 days

ii. Did cracked nipples or mastitis require treatment? Yes No

iii. If yes, type of treatment

a. Antibiotics

b. Incision and drainage

c. Other

4. Disclosure (for those who have not disclosed yet)

Since your last study visit, have you newly disclosed your status?

i. Yes ii. No

If yes, what is your relationship with that person?

1. Spouse
2. A parent
3. A child
4. A sibling
5. A friend
6. Grandparent
7. Other (state it**)......**

**5. 3-item self-report measure for Medication Adherence**

a. In the last 30 days, how many *days* did you miss at least one dose of any of your ARVS?

Write in the number of days: ____ (0–30)

b. In the last 30 days, how good a job did you do at taking your ARVs in the way you were supposed to?

1. Very poor
2. Poor
3. Fair
4. Good
5. Very good
6. Excellent

c. In the last 30 days, how often did you take your ARV in the way you were supposed to?

1. Never
2. Rarely
3. Sometimes
4. Usually
5. Almost always
6. Always

**Data extraction form at 6 months**

1. Mother’s viral load result at 6 months……………………………………….(copies/ml).

2. Infant’s current health status (circle one)

i. Alive and well Alive but unwell Dead

ii. If unwell, what is the diagnosis?

1. Pneumonia
2. TB
3. Gastroenteritis
4. Sepsis
5. Malaria
6. Prolonged jaundice
7. Other (please state)
8. Unknown

iii. Has there been any interval illness? (Illness since last seen and this visit)

Yes No

iv. If yes, what was the diagnosis?

1. Pneumonia
2. TB
3. Gastroenteritis
4. Sepsis
5. Malaria
6. Prolonged jaundice
7. Other (please state)
8. Unknown

v. If dead, record the cause of death from the infant’s medical records

**G. 12-MONTH VISIT**

Study Site: 1. Korle Bu Teaching Hospital 2. Ridge Regional Hospital

Date of interview

Study No:

1. Postpartum period (Number of weeks after delivery) ………………………………….

2. Type of Infant feeding (circle one)

1. Exclusive breastfeeding
2. Formula Feeding
3. Complementary Feeding Alone
4. Complementary feeding + breastmilk
5. Complementary feeds + formula
6. Complementary feeding + formula + breastmilk

3. Disclosure (for those who have not disclosed)

Since your last study visit, have you newly disclosed your status?

Yes No

If yes, what is your relationship with that person?

1. Spouse
2. A parent
3. A child
4. A sibling
5. A friend
6. Grandparent
7. Other (state it**)......**

**4. 3-item self-report measure for Medication Adherence**

a. In the last 30 days, how many *days* did you miss at least one dose of any of your ARVS?

Write in the number of days: ____ (0–30)

b. In the last 30 days, how good a job did you do at taking your ARVs in the way you were supposed to?

1. Very poor
2. Poor
3. Fair
4. Good
5. Very good
6. Excellent

c. In the last 30 days, how often did you take your ARV in the way you were supposed to?

1. Never
2. Rarely
3. Sometimes
4. Usually
5. Almost always
6. Always

**Data extraction form at 12 months**

1. Mother’s viral load result at 12 months ……………………………………….(copies/ml).

2. Infant’s current health status (circle one)

i. Alive and well Alive but unwell Dead

ii. If unwell, what is the diagnosis?

1. Pneumonia
2. TB
3. Gastroenteritis
4. Sepsis
5. Malaria
6. Prolonged jaundice
7. Other (please state)
8. Unknow

iii. Has there been any interval illness? (Illness since last seen and this visit)

Yes No

iv. If yes, what was the diagnosis?

1. Pneumonia
2. TB
3. Gastroenteritis
4. Sepsis
5. Malaria
6. Prolonged jaundice
7. Other (please state)
8. Unknown

v. If dead, record the cause of death from the infant’s medical records

3. Infant’s PCR result at 9 months……………………………………….

**H. Assessment of depressive symptoms at 6-8 weeks post-partum**

**A. THE EDINBURGH POSTNATAL DEPRESSION SCALE**

As you recently had a baby, we would like to know how you are feeling now. In the past 7 days:

**1. I have been able to laugh and see the funny side of things**

0 ☐ As much as I always could 1 ☐ Not quite so much now 2 ☐ Definitely not so much now 3 ☐ Not at all

**2. I have looked forward with enjoyment to things**

0 ☐ As much as I ever did 1 ☐ Rather less than I used to 2 ☐ Definitely less than I used to 3 ☐ Hardly at all

**3. I have blamed myself unnecessarily when things went wrong**

3 ☐ Yes, most of the time 2 ☐ Yes, some of the time 1 ☐ Not very often 0 ☐ No, never

**4. I have been anxious or worried for no good reason**

0 ☐ No, not at all 1 ☐ Hardly ever 2 ☐ Yes, sometimes 3 ☐ Yes, very often

**5. I have felt scared or panicky for no very good reason**

3 ☐ Yes, quite a lot 2 ☐ Yes, sometimes 1 ☐ No, not much 0 ☐ No, not at all

**6. Things have been getting on top of me**

3 ☐ Yes, most of the time I haven’t been able to cope 2 ☐ Yes, sometimes I haven’t been coping as well as usual 1 ☐ No, most of the time I have coped quite well 0 ☐ No, I have been coping as well as ever

**7. I have been so unhappy that I have had difficulty sleeping**

3 ☐ Yes, most of the time 2 ☐ Yes, sometimes 1 ☐ Not very often 0 ☐ No, not at all

**8. I have felt sad or miserable**

3 ☐ Yes, most of the time 2 ☐ Yes, quite often 1 ☐ Not very often 0 ☐ No, not at all

**9. I have been so unhappy that I have been crying**

3 ☐ Yes, most of the time 2 ☐ Yes, quite often 1 ☐ Only occasionally 0 ☐ No, never

**10. The thought of harming myself has occurred to me**

3 ☐ Yes, quite often 2 ☐ Sometimes 1 ☐ Hardly ever 0 ☐ Never

Date Completed: Total Score: / 30 Total Score for Question 10: / 3

Note: Question 10 is a single screening question on suicide risk. A patient who answers Yes, quite often or Sometimes, will be assessed further for suicide risk by the onsite clinical psychologist.
